# Automatic inhibition and habitual control: alternative views in neuroscience research on response inhibition and inhibitory control

**DOI:** 10.3389/fnbeh.2013.00025

**Published:** 2013-04-04

**Authors:** Agnes J. Jasinska

**Affiliations:** National Institute on Drug Abuse, Intramural Research Porgram, Neuroimaging Research BranchBaltimore, MD, USA

Decades of theory and experimental evidence underscore the critical importance of inhibitory functions to flexible, context-appropriate and goal-directed action (Diamond et al., 1963; Logan and Cowan, 1984; Aron et al., 2004; Friedman and Miyake, 2004; Ridderinkhof et al., 2004; Munakata et al., 2011). Deficits in inhibitory control and response inhibition have been implicated in substance use disorders, attention-deficit/hyperactivity disorder, and impulse control disorders (Jentsch and Taylor, 1999; Nigg, 2001; Li and Sinha, 2008; Groman et al., 2009), and may also play a role in depression and anxiety (Disner et al., 2011; Jovanovic and Norrholm, 2011). But despite exciting progress in this area of cognitive and clinical neuroscience, some fundamental questions remain unsolved, and may be hindering the translational efforts aiming at improving the treatment and prevention of disorders characterized by impairment or dysregulation of inhibitory functions.

The purpose of this Opinion is to highlight some alternative ideas and approaches in neuroscience research on response inhibition and inhibitory control that have emerged in the literature, in order to stimulate debate and suggest hypotheses for future research. In particular, building on recent neuroscience-based accounts of response inhibition (e.g., Mostofsky and Simmonds, 2008; van Gaal et al., 2008; Munakata et al., 2011), I argue *against* the long-standing and pervasive view that there is a fundamental distinction between inhibitory processes on the one hand and response selection and execution processes on the other hand. Although intuitive, this view does not appear to be supported by evidence. Instead, until there is evidence to the contrary, I propose a more parsimonious view of inhibitory control mechanisms in the brain, such that: (1) response inhibition can be either a control process (to override a prepotent response tendency) or it can itself be a prepotent response tendency (to be overridden); (2) response inhibition processes and response selection and execution processes are fundamentally the same; and (3) we learn to inhibit a response in fundamentally the same way that we learn to select and execute a response.

## Proposition 1: response inhibition can be either a control process (to override a prepotent response tendency) or a prepotent response tendency (to be overridden)

A long-standing assumption has been that response inhibition processes, and inhibitory control more generally, are fundamentally different from response selection and execution processes. This assumption in part derives from the centuries-old and highly influential tradition of dichotomizing between the top-down, voluntary and deliberate control processes, and the bottom-up, stimulus-driven and automatic response tendencies that often need to be overridden. In fact, a typical experimental paradigm used to examine inhibitory control (such as Go/NoGo and Stop-Signal tasks) requires a deliberate, effortful inhibition of a prepotent, automatic response, creating the illusion that inhibitory control is always deliberate and effortful whereas the response to be inhibited is always prepotent and automatic. But one can think of conditions and situations in which this relationship is reversed, e.g., a patient with social anxiety may have to intentionally and effortfully overcome his or her prepotent tendency to refrain from public speaking, and many of us would face a similar challenge if asked to get up on the stage and sing.

Therefore, I argue that response inhibition is not a control process *by default*; instead, it may function as either a control process (to override a prepotent response tendency) or as a prepotent response tendency itself (to be overridden), depending on the situational demands and/or the relative strengths of the two competing goal representations. I expand on the idea of inhibitory goal representation in Proposition 2, and I discuss the notion of automatic inhibition in Proposition 3.

## Proposition 2: the brain processes mediating response inhibition are fundamentally the same as the brain processes mediating response selection and execution

Evidence from human functional MRI (fMRI) studies using Go/NoGo, Stop-Signal, and similar tasks suggests that inhibition of a motor response engages the same network (or networks) of brain regions that are engaged during selection and execution of this motor response. In particular, response inhibition processes engage a distributed network of both cortical and subcortical regions, including the inferior frontal gyrus (IFG; or inferior frontal cortex, IFC), insula, anterior cingulate cortex (ACC), pre-supplementary motor cortex (pre-SMA), dorsolateral prefrontal cortex (DLPFC), parietal regions, and basal ganglia [e.g., (Rubia et al., 2001; Garavan et al., 2002; Aron and Poldrack, 2006); for meta-analyses, see (Wager et al., 2005; Simmonds et al., 2008; Swick et al., 2011; Criaud and Boulinguez, 2013; Hart et al., 2013)]. For instance, there is a compelling neuroimaging and lesion evidence of the critical role of the IFG in inhibitory control (Konishi et al., 1999; Aron et al., 2003; Rubia et al., 2003). Yet, as demonstrated by a recent fMRI study in over 1800 subjects (Whelan et al., 2012), a frontal network centered on the IFG is engaged both during successful inhibition trials and during failed inhibition trials (i.e., when subjects erroneously executed the response). Similarly compelling is the neuroimaging evidence for the importance of the pre-SMA in inhibitory control [e.g., (Rubia et al., 2001; Mostofsky et al., 2003; Garavan et al., 2006), for meta-analyses, see (Simmonds et al., 2008; Swick et al., 2011; Criaud and Boulinguez, 2013)]. But as reviewed by Mostofsky and Simmonds (2008), the pre-SMA also plays a critical role in both response preparation and response selection. In fact, evidence from electrophysiological recordings in non-human primates suggests that some pre-SMA neurons participate both in the suppression of the incorrect response and in the facilitation of the correct response in a saccade Go/NoGo task (Isoda and Hikosaka, 2007), suggesting that response inhibition processes overlap with response selection processes not only at the level of large-scale brain networks involved, but also at the level of individual neurons.

Similarly, at the level of synaptic transmission, response inhibition processes may not be fundamentally different from response selection and execution processes. It is sometimes assumed that response inhibition must rely on inhibitory synaptic transmission to a larger degree than response selection and execution processes. But why should it be the case? Inhibitory synaptic transmission involving the neurotransmitter gamma-aminobutyric acid (GABA) is known to be as important as excitatory glutamatergic transmission both at cortical and subcortical levels (Kandel et al., 2000). Fast inhibitory synaptic transmission is mediated primarily by ionotropic GABA_A_ receptors, which hyperpolarize the cell and thus raise the threshold for firing an action potential when activated. When GABA_A_ receptors are localized to postsynaptic glutamatergic neurons, they serve to inhibit the activity of these neurons. But GABA_A_ receptors can also be localized to postsynaptic GABAergic neurons, in which case they may serve to disinhibit (rather than inhibit) the activity of downstream glutamatergic neurons, leading to activation rather than inhibition at the local-circuit or larger-network level. Conversely, activation of glutamatergic neurons may be required to activate a group of GABAergic neurons and trigger inhibition. Thus, successful response inhibition likely relies on reciprocal interactions between inhibitory GABAergic neurons and excitatory glutamatergic neurons, rather than on inhibitory GABAergic transmission alone.

## Proposition 3: we learn to inhibit a response in fundamentally the same way than we learn to select and execute a response

We have a working model of how the brain learns to select and execute a specific response: such learning is thought to involve the formation of a distributed neural goal or task representation in the prefrontal cortex (Miller and Cohen, 2001; Sakai, 2008), by which a specific pattern of sensory input becomes progressively associated with a specific pattern of motor output via long-term potentiation (LTP) and associated synaptic-plasticity processes at glutamatergic synapses (Kandel et al., 2000). In comparison, the processes underlying inhibitory goal representations and inhibitory learning remain less well-understood—including synaptic plasticity at inhibitory synapses (for a recent review, see Castillo et al., 2011).

Nevertheless, if response inhibition processes are fundamentally the same as response selection and execution processes, then it follows that the neural representation of inhibitory goals (or Stop goals) should not fundamentally differ from the neural representations of response selection and execution goals (or Go goals), and the underlying learning processes should also be fundamentally the same. In the influential horse-race models (Logan and Cowan, 1984; Verbruggen and Logan, 2009b), response inhibition in a Stop-Signal task is conceptualized as a race between a Go process triggered by a Go stimulus (i.e., a Go goal) and a Stop process triggered by a Stop stimulus (i.e., a Stop goal). In this model, the competing Go and Stop goals are regarded as equivalent, and it is the relative timing of the Go and Stop processes that determines whether the response is successfully inhibited or not. In fact, Munakata and colleagues (2011) have argued that at least some inhibitory control processes can be understood in terms of such competition between goal representations in the prefrontal cortex, and it is the relative strength of these goal representations that determines whether a behavioral response is executed or inhibited by that individual in a given situation.

Furthermore, although counterintuitive, growing evidence suggests that response inhibition processes may be stimulus-driven to the same extent that response selection and execution processes are stimulus-driven. In fact, one and the same stimulus can activate both a goal representation to carry out a behavior and a goal representation to inhibit this behavior. For instance, food-related cues may activate both a goal to consume the food and a competing goal to stay on a diet (Fishbach et al., 2003; Hare et al., 2009; Kroese et al., 2011); smoking-related cues and smoking-cessation messages may activate both a goal to smoke a cigarette and a competing goal to abstain from smoking (Brody et al., 2007; Jasinska et al., 2012); and signals of threat may activate both aggressive and fear-related behaviors (Beaver et al., 2008; Passamonti et al., 2008).

Finally, following the same logic, there is no reason why response inhibition should not become automatic—and inhibitory control habitual—with appropriate and sufficient practice. Specifically, if response inhibition can be triggered by a Stop stimulus in the same fashion that response selection and execution is triggered by a Go stimulus, then a consistent mapping between a specific Stop stimulus and the inhibition of a specific response should result in practice-related improvements and eventually automaticity (Verbruggen and Logan, 2009a; Lenartowicz et al., 2011), even if Stop stimuli are not consciously perceived [(van Gaal et al., 2008, 2009, 2010); see also (Eimer and Schlaghecken, 2003)]. Indeed, such practice-related improvements have been demonstrated in the Go/NoGo task, which relied on consistent stimulus-inhibition associations, but not in the Stop-Signal task, in which no such associations were learned [(Verbruggen and Logan, 2008); see also, (Verbruggen and Logan, 2009b)]. Converging evidence of such learned stimulus-inhibition association—or *automatic inhibition*—following training in a Go/NoGo task was also demonstrated by reduced corticospinal excitability on Go trials preceded by NoGo trials, relative to Go trials preceded by Go trials (Chiu et al., 2012). Interestingly, despite a lack of such consistent stimulus-inhibition mapping, direct activation of the right IFG with transcranial direct current stimulation (tDCS) improved response inhibition in the Stop-Signal task relative to sham condition (Jacobson et al., 2011). These findings support the view that inhibitory control can become habitual. However, if response inhibition and response execution are learned in the same manner, it follows that learning of stimulus-inhibition associations should follow the same principles of initial specificity and subsequent generalization. These principles may determine the extent of generalization—or conversely, the limits of transfer—in training-based interventions for inhibitory control deficits.

## Conclusions

In this Opinion article, drawing on recent neuroscience-based accounts of inhibitory control mechanisms in the human brain (e.g., Mostofsky and Simmonds, 2008; van Gaal et al., 2008; Munakata et al., 2011), I argued that: (1) response inhibition can be either a control process (to override a prepotent response tendency) or it can itself be a prepotent response tendency (to be overridden); (2) response inhibition processes and response selection and execution processes are fundamentally the same; and (3) we learn to inhibit a response in fundamentally the same way that we learn to select and execute a response. These propositions have implications for both basic and translational neuroscience research on inhibitory control, and the goal is to stimulate debate and inspire novel hypotheses for future research, ultimately aimed at treatment and prevention of inhibitory control deficits.
